# Remediation of Nitrobenzene Contaminated Soil by Combining Surfactant Enhanced Soil Washing and Effluent Oxidation with Persulfate

**DOI:** 10.1371/journal.pone.0132878

**Published:** 2015-08-12

**Authors:** Jingchun Yan, Weiguo Gao, Linbo Qian, Lu Han, Yun Chen, Mengfang Chen

**Affiliations:** 1 Key Laboratory of Soil Environment and Pollution Remediation, Institute of Soil Science, Chinese Academy of Sciences, Nanjing, 210008, China; 2 University of Chinese Academy of Sciences, Beijing, 100049, China; Brandeis University, UNITED STATES

## Abstract

The combination of surfactant enhanced soil washing and degradation of nitrobenzene (NB) in effluent with persulfate was investigated to remediate NB contaminated soil. Aqueous solution of sodium dodecylbenzenesulfonate (SDBS, 24.0 mmol L^-1^) was used at a given mass ratio of solution to soil (20:1) to extract NB contaminated soil (47.3 mg kg^-1^), resulting in NB desorption removal efficient of 76.8%. The washing effluent was treated in Fe^2+^/persulfate and Fe^2+^/H_2_O_2_ systems successively. The degradation removal of NB was 97.9%, being much higher than that of SDBS (51.6%) with addition of 40.0 mmol L^-1^ Fe^2+^ and 40.0 mmol L^-1^ persulfate after 15 min reaction. The preferential degradation was related to the lone pair electron of generated SO_4_•^−^, which preferably removes electrons from aromatic parts of NB over long alkyl chains of SDBS through hydrogen abstraction reactions. No preferential degradation was observed in •OH based oxidation because of its hydrogen abstraction or addition mechanism. The sustained SDBS could be reused for washing the contaminated soil. The combination of the effective surfactant-enhanced washing and the preferential degradation of NB with Fe^2+^/persulfate provide a useful option to remediate NB contaminated soil.

## Introduction

Widespread use and improper disposal of organic pollutants such as nitrobenzene (NB) have resulted in the persistent soil contamination, which has been a major environmental issue because of its adverse impacts on human health and potential risks to the ecosystem [1]. For the remediation of NB contaminated soil, technologies such as bioremediation, phytoremediation, solidification/stabilization, chemical treatment have been developed in recent years [2–5]. However, the above technologies have limitations and the application of a single remediation technology cannot efficiently remove NB from contaminated soil. For example, bioremediation eliminates NB at low costs, however, it may be constrained by selection of active microbes and appropriate soil conditions suitable for microbial growth [6,7], and solidification/stabilization of NB using cement may lead to secondary pollution owing to the incomplete treatment. Therefore, physical and chemical remediation has received much attention due to its potential effectiveness in the complete degradation and mineralization of NB [8].

Among physical and chemical remediation techniques, surfactant enhanced remediation is a suitable approach for removal of hydrophobic organic pollutant [9,10]. The use of surfactant results in enhanced solubilization capabilities of micelles and decreased interfacial tension between the washing liquid and the soil particles, which facilitate the desorption of organic contaminants from soil pores [11,12]. Due to low critical micellar concentration (*c*_cmc_) of nonionic surfactants and weak tendency of anionic surfactants adsorption onto soil particles, nonionic or/and anionic surfactants are preferred for soil washing [13,14]. Because surfaces of soil particles are usually negatively charged, cationic surfactants are less used since they tend to be adsorbed strongly onto the soil at neutral pH [15]. The surfactant enhanced washing process may be efficient, but the pollutants can only be transferred from soil to washing effluent, further treatment is usually required for complete detoxification of the pollutant in the washing effluent. The presence of surfactant increases the difficulty in the post-treatment of pollutant, and the degradation treatment of the washing effluent may lead to inefficient use of surfactant. Therefore, it is a challenge to develop a new method for preferential degradation of NB in washing effluent during the remediation process of NB contaminated soils.

Persulfate (S_2_O_8_^2−^, *E* ^0^ = 2.01 V) is an oxidant (Eq 1), because its activation generates strong oxidizing species of sulfate anion radicals (SO_4_•^−^, *E* ^0^ = 2.6 V), advanced oxidation processes (AOPs) with persulfate are alternative to remediate contaminated soil and groundwater [16,17]. AOPs are based on SO_4_•^−^, which can be produced from persulfate activation through both physical and chemical modes (Eq 2 and Eq 3) [18]. The physical activations depend on external energy, which include heating, light, gamma and microwave irradiation, and the chemical activations of persulfate are generally carried out by using transition metal ions (Me, such as Fe^2+^, Cu^+^ and Ag^+^) as activators. Once SO_4_•^−^ is generated, it will react with contaminants through electron transfer process to form sulfate (Eq 4).

$${\text{S}}_{2}{\text{O}}_{8}{}^{2-}+\;2{\text{e}}^{-}\to 2\text{S}{\text{O}}_{4}{}^{2-}\;{\text{E}}^{0}=2.01\text{V}$$ (1)

$${\text{S}}_{2}{\text{O}}_{8}{}^{2-}+\text{thermal},\;\text{microwave or UV}\to 2\text{S}{\text{O}}_{4}{•}^{-}$$ (2)

$${\text{S}}_{2}{\text{O}}_{8}{}^{2-}+\text{M}{\text{e}}^{\text{n}+}\to \text{M}{\text{e}}^{(\text{n}+1)+}+\text{S}{\text{O}}_{4}{•}^{-}+\text{S}{\text{O}}_{4}{}^{2-}$$ (3)

$$\text{S}{\text{O}}_{4}{•}^{-}+{\text{e}}^{-}\to \text{S}{\text{O}}_{4}{}^{2-}\;{\text{E}}^{0}=2.6\text{V}$$ (4)

Compared with hydroxyl free radical (•OH), SO_4_•^−^ has a longer lifespan with half life of 4 s in aqueous solution to interact with target pollutants, which can maintain effective degradation efficiency and high utilization of persulfate. Above all, due to the lone pair electron of SO_4_•^−^, electron transfer reactions are preferred, whereas •OH can only participate in a variety of reactions through hydrogen abstraction or addition mechanism with equal preference [19–21]. Therefore, SO_4_•^−^ based oxidation is more efficient than •OH for the degradation of trifluoroacetic acid [22]. The strong oxidation ability and the electron transfer reactions of SO_4_•^−^ makes persulfate based AOPs effective for the degradation of recalcitrant organic compounds.

In this study, anionic surfactant sodium dodecylbenzene sulfonate (SDBS, *c*_cmc_ 1.2 mmol L^-1^) and nonionic surfactant Tween 80 (*c*_cmc_ 0.099 mmol L^-1^) were selected as washing agents to extract NB from contaminated soil samples. After washing, the effluent containing surfactant and NB was collected for further degradation treatment. The surfactants were themselves degraded and their aggregation structures could largely hinder the degradation of the target pollutants in •OH based oxidation systems due to nonselective character of •OH [23,24]. In contrast, SO_4_•^−^ may preferably remove electrons from aromatic NB from NB-surfactant mixtures in the washing effluent because of the lone pair electron of SO_4_•^−^. The surfactant might survive after preferential degradation of NB, and may be reused in successive washing processes. The obtained effluent was treated with Fe^2+^/persulfate system, in comparison with the •OH based oxidation system of Fe^2+^/H_2_O_2_. This study provided an alternative method to achieve efficient and preferential degradation of the target NB with the effect of SO_4_•^−^ in the washing effluent of the NB-surfactant mixtures, which is useful for the in situ remediation of NB contaminated soil.

## Materials and Methods

### Chemicals and materials

FeSO_4_·7H_2_O, SDBS, Tween 80, H_2_O_2_ and persulfate were purchased from Sinopharm Chemical (Shanghai, China). NB was obtained from Lingfeng Chemical Reagents Plant (Shanghai, China). Kaolin was used as the representative soil in laboratory experiments because of its low organic content, low cation exchange capacity and inertia properties [25]. Kaolin was provided by Shanghai Fengxian Fengcheng Chemical Reagent Factory (Shanghai, China), and has the physical and chemical properties as follows: particle size < 2 μm, 37–38% Al content, 0.28% organic content, 1.71 cmol/100 g cationic exchangeable capacity (determined by BaCl_2_-H_2_SO_4_ method (ISO 11260–1997)), 3.36 zero point of charge (ZPC). All the chemicals except of Kaolin were analytical grade reagents. Double distilled water was used in the experiment.

### Preparation of NB contaminated soil

The NB contaminated soil was prepared with following steps. NB solution was obtained by dissolving 5.0 mg NB in 100.0 mL methanol. The solution was slowly added into soil (100.0 g) with vigorous stir for about 30 min to promote homogeneous distribution of NB in soil phase. The resultant mixture was well mixed and placed in a ventilation hood at room temperature for 7 days to allow the complete evaporation of the methanol [26], resulting in the contaminated soil in dry state.

### Solubility enhancement of NB by SDBS and Tween 80

Solutions with a series of surfactant concentrations were placed in flask with a Teflon lined-cap, and NB was subsequently added to each flask in an amount slightly more than that being required to saturate the solution. The initial surfactant concentrations were set at various values below and above the nominal *c*_cmc_ of surfactants. Triplicate samples were prepared for each surfactant concentration and then equilibrated for 2 h after vibration on a reciprocating shaker at 150 rpm for 24 h. The NB concentrations in aqueous supernatant phases were analyzed by high performance liquid chromatography (HPLC).

### Sorption of SDBS and Tween 80 on soil

Sorption isotherm experiments were performed to determine the equilibrium partitioning behavior of surfactants by a conventional batch equilibration method [27], in which the soil (5.0 g) was well mixed with surfactant solutions (100.0 mL) at different concentrations (*c*_surf_) in centrifuge tubes with Teflon-lined caps. The centrifuge tubes were then gently shaken for 24 h at 25°C in dark (the time of 24 h was confirmed to be adequate for equilibration from preliminary studies). After shaking, the tubes were centrifuged at 8,000 rpm for 10 min to separate the water and solid phases. The supernatant was taken for analysis to determine the concentration of surfactants (*c*_e_) with HPLC. The adsorbed concentration of surfactant on soil was calculated by the difference between *c*_surf_ and *c*_e_.

### Soil remediation by surfactant solutions

The 5.0 g soil sample was placed in a 250 mL flask with a Teflon-lined cap, to which 100.0 mL of different washing solvents were added, and the flask was placed on a stirring apparatus for 2 h to ensure adsorption-desorption equilibrium. Two treatments were tested, including different concentrations of SDBS and Tween 80. Each treatment was replicated for three times. After washing, the soil suspension was centrifuged at 8,000 rpm for 10 min and the liquid phase was sampled and filtered through a 0.2 μm membrane for analysis. The concentrations of NB and surfactant in the liquid phase (the filtered solution) were determined.

### Treatment of washing effluent

The degradation of the obtained effluent containing NB and surfactant was carried out following the washing step. For each degradation experiment, the washing effluent was stirred in batch of vessel reactor (100 mL) at room temperature and pH 3.0 unless otherwise specified elsewhere. Typically, an appropriate amount of the oxidant (S_2_O_8_^2−^ or H_2_O_2_) to reach the desired concentrations was added to the effluent, and the initial concentrations (*c*_0_) of NB and SDBS were then measured. The reaction was initiated immediately by adding Fe^2+^. At regular time intervals, 2 mL of the solution was sampled, quenched with Na_2_S_2_O_3_ and the concentrations of NB and SDBS were measured, respectively.

### Analytical methods

The concentration of NB was analyzed by using JASCO HPLC equipped with UV-2075 plus intelligent UV/vis detector (Jasco, Japan) and a Prostar 420 auto flow injection system (Varian, America). An amethyst C_18_-P column (5 *μ*m, 4.6×250 mm) was used as separation column. The mobile phase used for HPLC experiment was a mixture of methanol and water (63:37, v/v) with a flow rate of 1.0 mL min^-1^, and the UV detector was operated at 285 nm.

The concentrations of SDBS and Tween 80 were also analyzed by HPLC equipped with UV/vis detector mentioned above. An amethyst C_18_-P column (5 *μ*m, 4.6×250 mm) was used as separation column. The mobile phase was a mixture of methanol/water with a flow rate of 1.0 mL min^-1^. The methanol/water (v/v) ratios and the detecting wavelengths for SDBS and Tween80 were 70:30, 238 nm and 90:10, 242 nm, respectively.

## Results and Discussion

### Recovery of NB adsorbed on soil

Because of the possible evaporation of NB with methanol in open-air drying processes, the preparation method for NB contaminated soil was evaluated by the recovery of NB. Tests for the NB recovery were started with random collections of the uniform contaminated soils, and extracted with methanol for 3 times. The average recovery of NB was 47.3 (±1.4) mg kg^-1^, and the value in parenthesis was standard deviation (n = 6). This value was used as the initial concentration of NB for all experiments.

### Apparent solubility of NB in SDBS and Tween 80 solution

**Fig 1:**
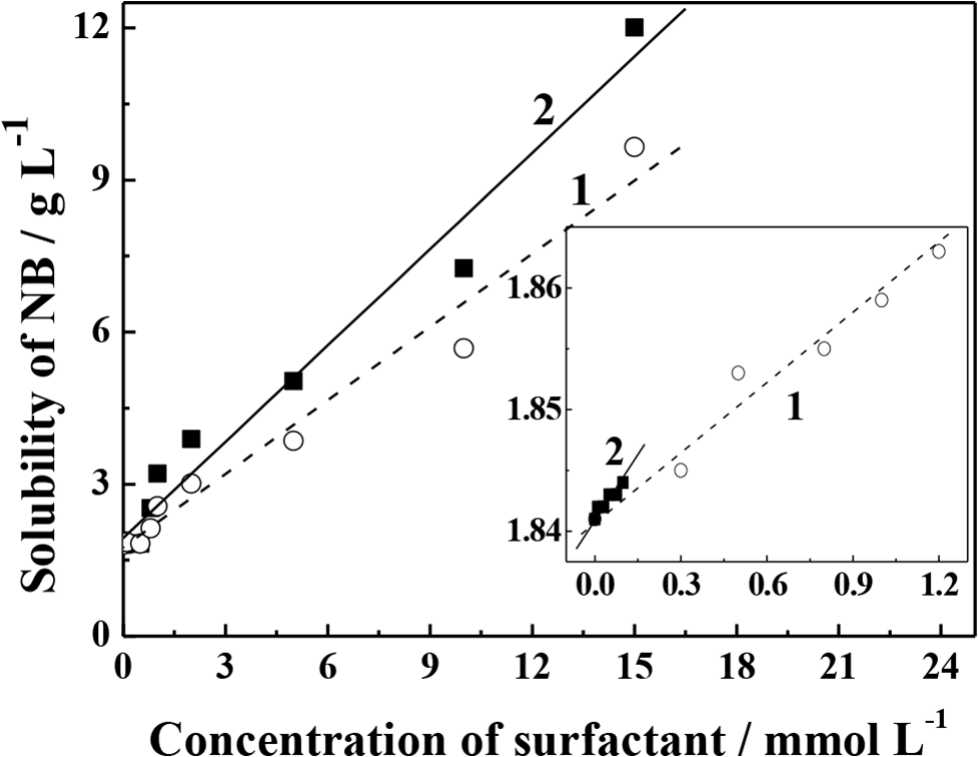
Apparent solubility enhancement of NB by (1) SDBS and (2) Tween 80.

Experiments were carried out to quantify the changes of NB solubility in aqueous solution as a function of the aqueous concentrations of SDBS and Tween 80, respectively. It is illustrated in Fig 1 in which data are bilinear in both cases of SDBS and Tween 80. When the surfactant concentration was less than *c*_cmc_, the apparent solubility of NB increased slowly with the increase of its concentration. As the surfactant concentration exceeded *c*_cmc_, the apparent solubility of NB increased significantly. The apparent solubility of NB in SDBS or Tween 80 solution is expressed as Eq 5.

$$S_{W}{}^{*}/S_{W}=\;1+X_{mn}K_{mn}+X_{mc}K_{mc}$$ (5)

Where *S*_W_* was the apparent NB solubility when NB or Tween 80 concentration was *X*; *S*_W_ was the NB solubility in water (1.8 g L^-1^ in our case); *X*_mn_ was the monomer form surfactant concentration (if *X* ≤ *c*_cmc_, *X*_mn_ = *X*; if *X* > *c*_cmc_, *X*_mn_ = *c*_cmc_); *X*_mc_ was the micellar form surfactant concentration (if *X* ≤ *c*_cmc_, *X*_mc_ = 0; if *X* > *c*_cmc_, *X*_mc_ = *X*- *c*_cmc_); *K*_mn_ was the partition coefficients of NB between surfactant monomer and water, and *K*_mc_ was the partition coefficients of NB between micelles and water [28].

It was illustrated that the sub-cmc slope (*K*_1_) and the supra-cmc slope (*K*_2_) obtaining from fitting the curves of NB solubility enhancement equations were NB partition coefficient between the surfactant monomer and the bulk aqueous, and between the surfactant micellar and the bulk aqueous, respectively [29,30]. Thus, Eq 6 and Eq 7 can be deduced based on Eq 5.

$$K_{mn}=K_{1}/S_{W}$$ (6)

$$K_{mc}=K_{2}/S_{W}$$ (7)

From Eq 6 and Eq 7, the values of *K*_mn_ and *K*_mc_ for NB in SDBS and Tween 80 solutions were calculated, giving 3.5 and 114.4 mL g^-1^ for SDBS, 59.4 and 1394.1 mL g^-1^ for Tween 80, respectively. It can be concluded that the water solubility of NB is increased both by surfactant monomer and micelles, and the solubility effect is significantly enhanced when the surfactant concentration is above its *c*_cmc_. However, the greater *K*_mc_ and *K*_mn_ values of Tween 80 than those of SDBS suggest that Tween 80 has greater affinity for NB.

### Adsorption of surfactants on soil

**Fig 2:**
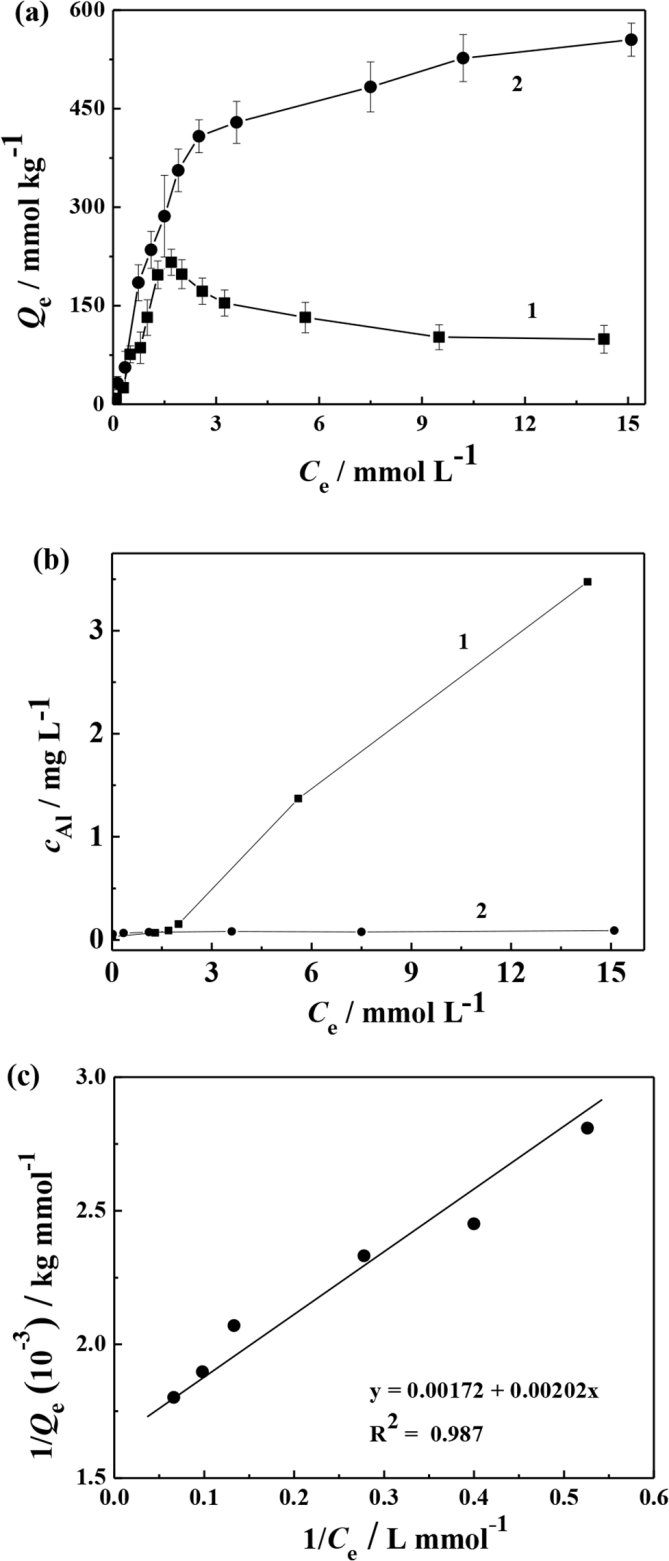
(a) Sorption isotherms of (1) SDBS (pH 6.5) and (2) Tween 80 (pH 6.8) on kaolin. (b) Concentrations of leached Al in (1) SDBS and (2) Tween 80 solutions at different concentrations. (c) The Langmuir sorption mode of Tween 80 on kaolin.

The adsorption of surfactants on soil is critical in evaluating NB desorption from soil, and the adsorption isotherms of SDBS and Tween 80 on soil were measured. For SDBS (curve 1 in Fig 2A), the adsorption amount of SDBS was sharply increased with the increase of SDBS concentrations ranging from 0 to 1.7 mmol L^-1^. The adsorption amount of SDBS achieved a maximum of 216.3 mmol kg^-1^ at the concentration of 1.7 mmol L^-1^. Further rise in the SDBS concentration above 1.7 mmol L^-1^ caused slow fall in the adsorption amount of SDBS.

The aqueous SDBS at lower SDBS concentration was present in the form of aqueous monomers since its concentrations were less than *c*_cmc_. Positive charged groups (e.g.,–NH_4_^+^ and–OH_2_^+^ groups and Al-oxides) were existed on the surfaces of soil particles, and SDBS might be adsorbed on the positively charged groups by electrostatic attraction [31]. When the SDBS concentrations in water were slightly higher than its *c*_cmc_ value, SDBS was adsorbed on the surfaces of soil particles due to the effect of its hydrophilic sulfonate group with its hydrocarbon chain orientated towards the solution [32]. As SDBS concentrations were further increased, the interactions of hydrocarbon chains between the adsorbed SDBS and the dissolved SDBS in solution drove the formation of a bilayer, which favored further adsorption of SDBS on soil [33]. At higher SDBS concentrations, the decrease of adsorption of SDBS on soil may be due to the mineral dissolution or precipitation of soil. In order to validate it, the leached Al in SDBS solution under the above conditions was examined because the main minerals in kaolin contain Al. It was found that the amount of leached Al in SDBS solution was increased with the increase of SDBS concentrations (Fig 2B). It suggests that the dissolution of Al-contained minerals in SDBS led to a decreased adsorption amount of SDBS on soil. These phenomena were agreed with the adsorption isotherms of SDBS on kaolinite and montmorillonite observed by Torn et al. [34] and Yang et al. [35], respectively.

Unlike SDBS, no mineral dissolution of soil was observed in the case of Tween 80, and the shape of its adsorption isotherm is different from that of SDBS. The isotherm of Tween 80 adsorption was well fitted to Langmuir sorption model (Eq 8),

$$1/Q_{e}=1/Q_{m}+1/KQ_{m}\times \;1/C_{e}$$ (8)

where *Q*_e_ is the amount of adsorbed Tween 80 in soil phase (mmol kg^−1^), *K* is the adsorption constant of Tween 80, and *Q*_m_ is the maximum amount of adsorbed Tween 80 in soil phase (mmol kg^−1^). The Langmuir sorption mode of Tween 80 on kaolin was plotted in Fig 2C, and *Q*_m_ was calculated to be 581.3 mmol kg^−1^. The adsorption amount of Tween 80 is higher than that of SDBS on soil is unfavorable for NB desorption during the washing process, because the dissolved NB in Tween 80 adsorbed on soil is difficult to be washed out.

### Soil washing performances with surfactants

**Fig 3:**
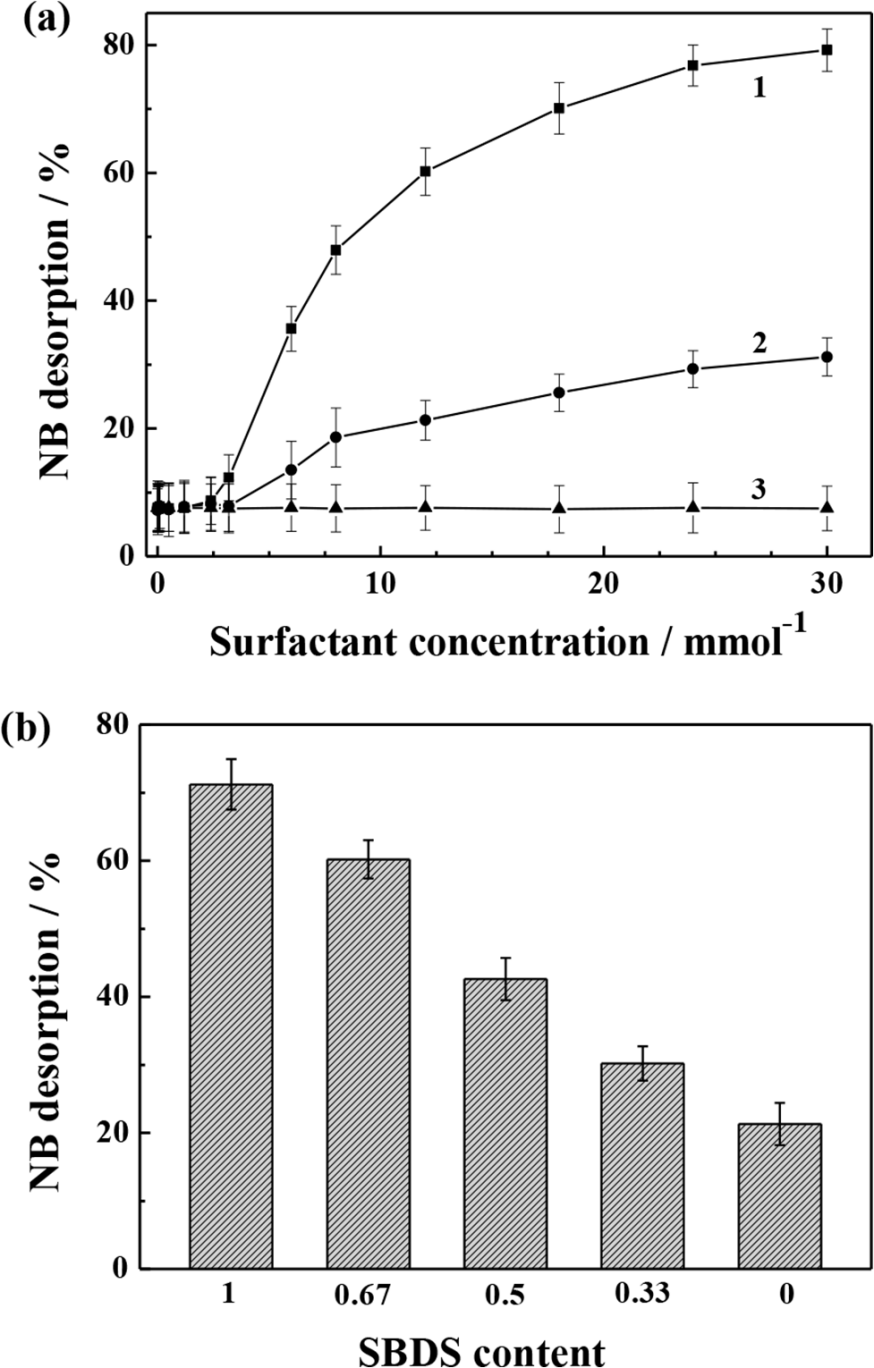
(a) Desorption of NB from kaolin in the presence of (1) SDBS, (2) Tween 80, and (3) no any surfactant except of distilled water. (b) Effect of the composition of the mixed surfactants (SDBS + Tween 80) with total concentration of 24.0 mmol L^-1^. SDBS content = 1 and SDBS content = 0 in (b) referred to single SDBS and Tween 80, respectively.

The desorption of NB from kaolin in the presence of water, single surfactant of SDBS and Tween 80 were conducted. With the addition of water, only 7.6% of NB desorption efficiency was achieved in accordance with the hydrophobic property of NB. When the SDBS concentration was below 2.4 mmol L^-1^ or the concentration of Tween 80 was below 3.2 mmol L^-1^, the desorption efficiency of NB was not obviously increased with the increase of individual surfactant concentration (Fig 3A). At such concentrations, SDBS and Tween 80 were mainly adsorbed onto kaolin, SDBS and Tween 80 in aqueous phase were present in the form of monomers since their aqueous concentrations were less than their *c*_cmc_. With subsequently increasing of the surfactant concentration, the NB desorption efficiency was correspondingly increased, reaching 76.8% and 29.3% with the addition of 24.0 mmol L^-1^ SDBS and Tween 80, respectively. Further rise in their concentrations resulted in only slight increase in NB desorption efficiency.

When the surfactant concentration was less than 2.4 mmol L^-1^ for SDBS or 3.2 mmol L^-1^ for Tween 80 in washing solution, the surfactants were almost adsorbed on kaolin. As the surfactant concentration was further increased but below *c*_cmc_, NB in the liquid phase was more easily trapped in the hydrophobic cores of the surfactant monomer, and only the molecules of NB transferred into internal micelle could be washed out [36,37]. Such transfer only took place within the aqueous phase. Because only a small amount of NB was in aqueous phase due to the hydrophobic properties of NB, effective NB desorption rarely occurred under this condition. When the concentration of the surfactant was increased beyond the *c*_cmc_, the micellar concentration was increased progressively, the NB remained in the liquid phase was reduced, which allowed a better chance of the fixed NB molecules interacting with the unoccupied micelles. The fixed NB molecules on the soil were the only source extracted by surfactant micelles. Therefore, a rapid desorption efficiency of NB on soil was observed, along with a monotonous rise of the washing performance curve. As the surfactant concentration was at a relatively high level (24.0 mmol L^-1^), most of the NB molecules had been dissolved in the micellar phase. Due to the adsorption effect of surfactant onto soil, further increasing surfactant concentration could only lead to a slow increase in the NB desorption efficiency. Compared with Tween 80, SDBS yielded higher desorption efficiency of NB at the same concentration due to the weaker sorption effect on kaolin, though the apparent solubility of NB in Tween 80 solution was greater than that in SDBS solution. It can be concluded that the adsorption amount of surfactant on soil is the key in washing process, which can significantly affect NB desorption.

Desorption of NB from kaolin was also conducted by using the mixtures of SDBS and Tween 80 with a fixed total concentration of 24.0 mmol L^-1^. It was demonstrated that the desorption of NB with the SDBS-Tween 80 mixture was higher than that with single Tween 80 (SDBS content = 0), but lower than that with single SDBS (SDBS content = 1). In order to achieve higher NB degradation efficiency with lower economic costs, 100.0 mL surfactant solution with 24.0 mmol L^-1^ SDBS was selected for washing kaolin (5.0 g) contaminated by NB (47.3 mg kg^-1^). After washing, the effluent was collected and subsequently subjected for the treatment. Analytical results demonstrated the effluent containing 0.015 mmol L^−1^ NB and 9.4 mmol L^−1^ SDBS.

### Treatment of washing effluent

**Fig 4:**
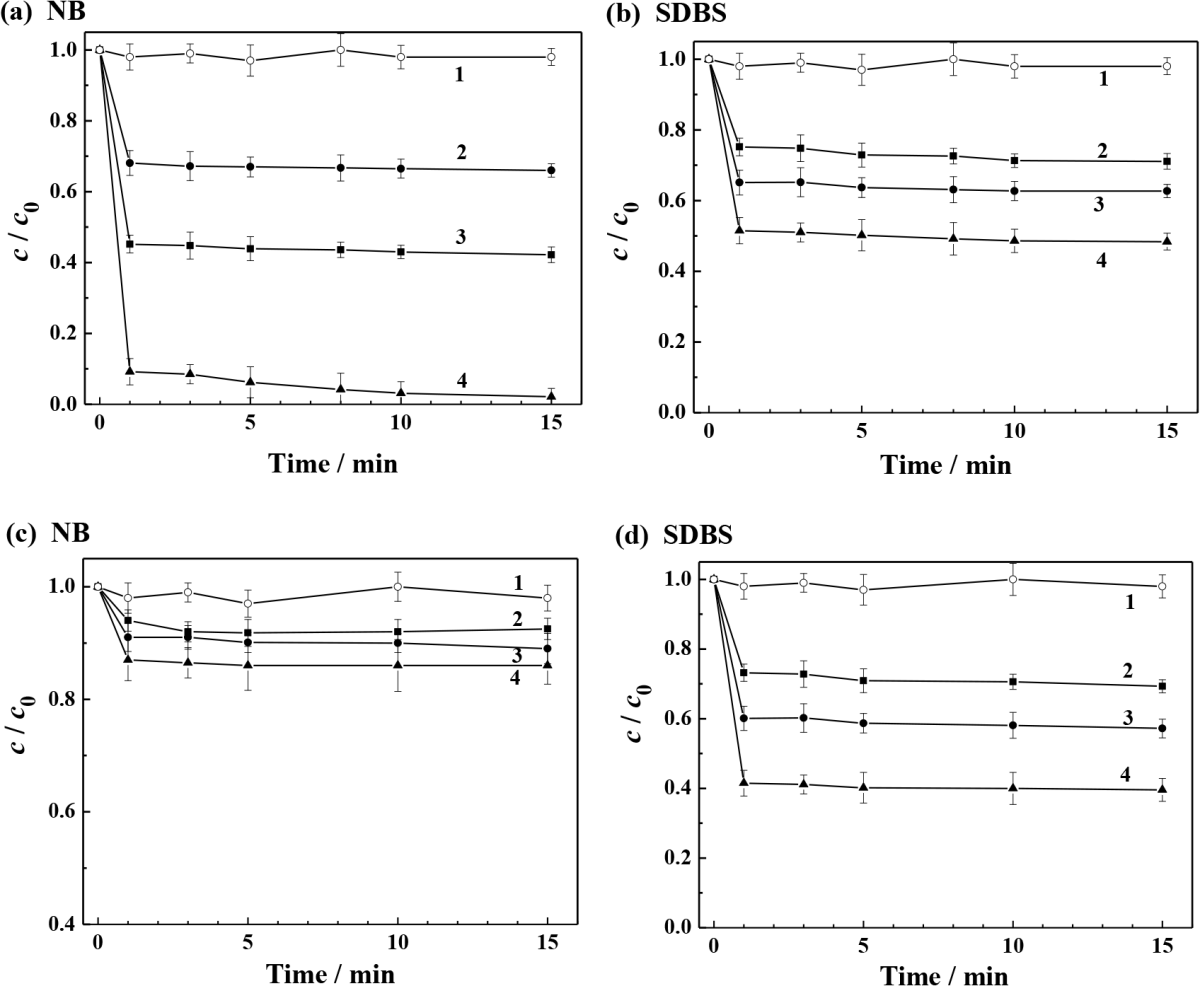
Degradation profiles of NB and SDBS in the washing effluent with the addition of H_2_O_2_ or persulfate. Degradation conditions in (a) and (b) were (1) 20.0 mmol L^-1^ S_2_O_8_^2−^, (2) 20.0 mmol L^-1^ Fe^2+^ + 20.0 mmol L^-1^ S_2_O_8_^2−^, (3) 40.0 mmol L^-1^ Fe^2+^ + 40.0 mmol L^-1^ S_2_O_8_^2−^, and (4) 80.0 mmol L^-1^ Fe^2+^ + 80.0 mmol L^-1^ S_2_O_8_^2−^. Degradation conditions in (c) and (d) were (1) 20.0 mmol L^-1^ H_2_O_2_, (2) 20.0 mmol L^-1^ Fe^2+^ + 20.0 mmol L^-1^ H_2_O_2_, (3) 40.0 mmol L^-1^ Fe^2+^ + 40.0 mmol L^-1^ H_2_O_2_ and (4) 80.0 mmol L^-1^ Fe^2+^ + 80.0 mmol L^-1^ H_2_O_2_. The effluent composition: NB 0.015 mmol L^−1^, SDBS 9.4 mmol L^−1^, and pH 3.0.

For further detoxification of the target compound of NB, the washing effluent containing NB and SDBS was treated within the Fe^2+^/S_2_O_8_^2−^ system (Fig 4). When only S_2_O_8_^2−^ was added in the effluent, no noticeable removals of NB and SDBS were observed. With further addition of Fe^2+^, SO_4_•^−^ radicals were generated effectively, which resulted in the degradation of NB and SDBS proceeding quickly.

In the Fe^2+^/S_2_O_8_^2−^ system, the degradation of NB under the tested conditions proceeded rapidly within the initial 1 min, and then became slow subsequently. These indicated that the activated decomposition of S_2_O_8_^2−^ by Fe^2+^ was rapid as a large number of SO_4_•^−^ radicals were generated transiently once Fe^2+^ ions contacted with S_2_O_8_^2−^ anions. Preliminary experiments on the effect of the molar ratio of Fe^2+^ to S_2_O_8_^2−^ on NB removal were undertaken giving the optimum molar ratio of Fe^2+^ to S_2_O_8_^2−^ of 1:1. Such optimum molar ratio is in accordance with that (1:1) for persulfate activated by Fe^2+^ in trichloroethylene degradation and for Fe^2+^ to H_2_O_2_ when treating methyl tertiary butyl ether in Fenton system reported by Liang et al. [19] and Burbano et al. [38], respectively. In the case of 20.0 mmol L^-1^ Fe^2+^ and 20.0 mmol L^-1^ S_2_O_8_^2−^, the degradation efficiency of NB reached 34.0% along with SDBS degradation efficiency of 28.9%. By increasing the concentrations of both Fe^2+^ and S_2_O_8_^2−^ to 40.0 mmol L^-1^, the degradation efficiency achieved 57.8% and 37.3% for NB and SDBS, respectively. Further increase on the Fe^2+^ and S_2_O_8_^2−^ concentrations to 80.0 mmol L^-1^ resulted in almost complete NB degradation with removal efficiency of 97.9% in comparison with a SDBS removal efficiency of only 51.6%. By comparing the degradation kinetics and concentrations of NB and SDBS in the complex solutions, it was readily deduced that the reactivity of the oxidizing species of SO_4_•^−^ to the two compounds was significantly different. When the concentrations of Fe^2+^ and S_2_O_8_^2−^ were increased, the NB degradation removal rate was increased much faster than that of SDBS. It is indicated that the degradation of NB in the NB-SDBS solution is preferential over that of SDBS with the effect of SO_4_•^−^.

The NB-SDBS effluent was also treated in the Fe^2+^/H_2_O_2_ system involving •OH. It can be illustrated that the increase in the concentrations of Fe^2+^ and H_2_O_2_ could only result in higher SDBS degradation removal, but without obvious increase in the NB degradation removal. The data obtained here suggests that no preferential degradation in the NB-SDBS effluent occurred in the case of •OH.

Remediation of NB contaminated soil enhanced by the SDBS washing, gives a great advantage over degradation of the NB-SDBS effluent in the Fe^2+^/S_2_O_8_^2−^ system, because after NB was almost completely degraded at 97.9%, about half of the added SDBS was still remaining in solution. These provided a possibility for the reuse of the treated effluent for subsequent washing processes.

### Preferential degradation of NB in the NB-SDBS mixture

To illustrate the preferential degradation of NB in the NB-SDBS mixture by using SO_4_•^−^, solutions with different molar ratios of NB to SDBS were prepared and the degradation experiments were conducted in both Fe^2+^/S_2_O_8_^2−^ and Fe^2+^/H_2_O_2_ systems. The NB concentration was fixed at 0.1 mmol L^-1^, while the SDBS concentrations were varied at 0.1, 0.2 and 0.3 mmol L^-1^. The apparent rate constants of NB and SDBS degradation in Fe^2+^/S_2_O_8_^2−^ and Fe^2+^/H_2_O_2_ systems were calculated (S1 File and S1 Fig).

It can be demonstrated from Table 1 that the values of *k*_NB_ for the NB degradation in the NB-SDBS mixture by Fe^2+^/S_2_O_8_^2−^ are greater than that by Fe^2+^/H_2_O_2_. In contrast, *k*_NB_ for SDBS (*k*_SDBS_) in Fe^2+^/S_2_O_8_^2−^ is lower than that in Fe^2+^/H_2_O_2_. In the Fe^2+^/S_2_O_8_^2−^ system, following the increase in SDBS concentration, *k*_NB_ was slightly increased, but *k*_SDBS_ was decreased significantly. These led to a marked increase on the ratio of *k*_NB_/*k*_SDBS_, which was defined as selectivity coefficient (*R*) for the preferential degradation of NB from the NB-SDBS mixture. For example, in the mixture of 0.1 mmol L^-1^ SDBS and 0.1 mmol L^-1^ NB with the Fe^2+^/S_2_O_8_^2−^ system, *k*_NB_ at 1.40 min^-1^ is 5.4 times higher than *k*_SDBS_ at 0.26 min^-1^. When SDBS concentration was increased from 0.1 to 0.3 mmol L^-1^, *k*_NB_ was only slightly decreased from 1.403 to 1.282 min^-1^, but *k*_SDBS_ was markedly decreased from 0.257 to 0.072 min^-1^. Consequently, *R* was increased from 5.4 to 17.8. Due to R > 1, the above experimental results demonstrated that the Fe^2+^/S_2_O_8_^2−^ system can achieve preferential NB degradation in the NB-SDBS mixture, as evidenced by a relative lower concentration of NB in the mixture.

**Table 1:** Rate constants for degradation of NB and SDBS in their mixture by Fe^2+^/S_2_O_8_^2−^ and Fe^2+^/H_2_O_2_ systems. Reaction conditions: S_2_O_8_^2−^ 1.0 mmol L^−1^, H_2_O_2_ 1.0 mmol L^−1^, Fe^2+^ 1.0 mmol L^−1^, NB 0.1 mmol L^-1^ and pH 3.0.

*c*_SDBS_ (mmol L^-1^)	Fe^2+^/S_2_O_8_^2−^			Fe^2+^/H_2_O_2_		
	*k*_NB_ (min^-1^)	*k*_SDBS_ (min^-1^)	*R* ^a^	*k*_NB_ (min^-1^)	*k*_SDBS_ (min^-1^)	*R*
0.1	1.403	0.257	5.46	0.709	1.030	0.69
0.2	1.351	0.119	11.35	0.241	0.361	0.67
0.3	1.282	0.072	17.81	0.094	0.135	0.70

^a^ *R* = *k*_NB_/*k*_SDBS_

Unlike the Fe^2+^/S_2_O_8_^2−^ system, the Fe^2+^/H_2_O_2_ system did not show such preferential degradation of either NB or SDBS. In the Fe^2+^/H_2_O_2_ system, with the increase in SDBS concentration, both *k*_NB_ and *k*_SDBS_ were decreased, with the ratio of *k*_NB_/*k*_SDBS_ (*R* = 0.67–0.70) being remained unchanged. The observation for the preferential degradation of NB from the mixture by the Fe^2+^/S_2_O_8_^2−^ system rather than the Fe^2+^/H_2_O_2_ system may be attributed to different radicals generated in the two oxidation systems with different ways reacting with organic contaminants (NB and SDBS).

SO_4_•^−^ is an electrophilic reagent with lone pair electron, and prefers to participate in electron transfer reaction with aromatic compound to produce an organic radical cation when SO_4_•^−^ attacks organic compounds, though it can also react with alkane through hydrogen abstraction reactions [39,40]. In the mixture solution of aromatic compound NB and long alkyl chain compound SDBS, the generated SO_4_•^−^ preferably removes electrons from aromatic parts of NB over that from long alkyl chains of SDBS through hydrogen abstraction reactions, exhibiting preferential degradation of NB over SDBS. Due to the increasing SDBS concentration did not enhance markedly the competition of SDBS for SO_4_•^−^, which may explain that the favorable degradation of NB was increased with increasing SDBS concentration. When •OH attacks organic compounds, it is more likely to go through hydrogen abstraction or addition reactions with equal chances when reacting with NB and SDBS, resulting in no preferential oxidation of either NB or SDBS.

## Conclusions

A coupled process of surfactant enhanced soil washing followed by AOPs with persulfate was proposed for the remediation of NB contaminated soil. When 24.0 mmol L^-1^ of SDBS in the solution was used to extract soil contaminated by NB (47.3 mg kg^-1^), NB desorption efficiency of 76.8% was achieved, being superior to both Tween 80 and the SDBS/Tween 80 mixture. The washing effluent containing NB and SDBS was subsequently treated by the Fe^2+^/persulfate and Fe^2+^/H_2_O_2_ systems. It was found that the preferential degradation removal of NB was 97.9%, much higher than 51.6% for the degradation of SDBS after 15 min reaction with the addition of 40.0 mmol L^-1^ Fe^2+^ and 40.0 mmol L^-1^ persulfate. The preferential degradation is believed to be related to the lone pair electron of SO_4_•^−^, which preferably remove electrons from aromatic parts of NB compared with long alkyl chains of SDBS through hydrogen abstraction reactions. No preferential degradation was observed in •OH based oxidation because of its hydrogen abstraction or addition mechanism. The preferential degradation removal of NB from the NB-SDBS mixtures implies that the remained SDBS can potentially be reused for subsequent soil washing. Therefore, surfactant-enhanced washing and preferential degradation of NB from the resultant washing effluent with the Fe^2+^/persulfate system present a more practical option for the remediation of NB contaminated soil.

## Supporting Information

S1 Fig Degradation of NB (a) and SDBS (b) in a mixture of NB (0.1 mmol L^-1^) and SDBS (0.1 mmol L^-1^) by using a system of Fe^2+^ (1.0 mmol L^−1^) and S_2_O_8_^2−^ (1.0 mmol L^−1^) at pH 3.0. (TIF)

S1 File Determination of the apparent rate constants of NB and SDBS in Fe^2+^/S_2_O_8_^2−^ and Fe^2+^/H_2_O_2_ systems. (DOC)
