# Supplementary figures and images for: Remediation of Nitrobenzene Contaminated Soil by Combining Surfactant Enhanced Soil Washing and Effluent Oxidation with Persulfate
Source: PLoS One. 2015 Aug 12;10(8):e0132878. doi: 10.1371/journal.pone.0132878 (PMC4534389; doi:10.1371/journal.pone.0132878)

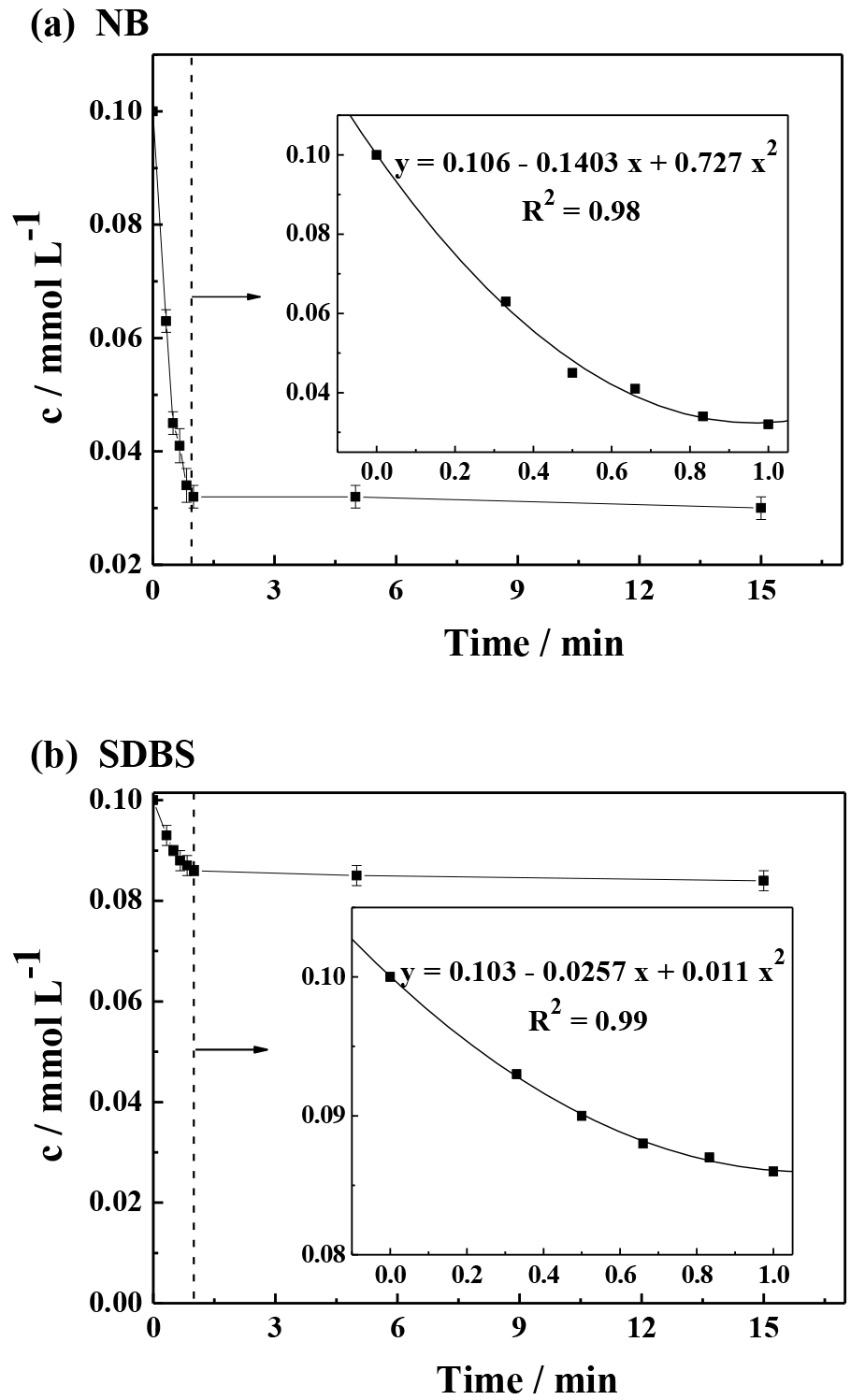

Supplement: S1 Fig — (TIF) [file pone.0132878.s001.tif]
