# Supplementary material for: Remediation of Nitrobenzene Contaminated Soil by Combining Surfactant Enhanced Soil Washing and Effluent Oxidation with Persulfate
Source: PLoS One. 2015 Aug 12;10(8):e0132878. doi: 10.1371/journal.pone.0132878 (PMC4534389; doi:10.1371/journal.pone.0132878)
Supplement: S1 File — (DOC) [file pone.0132878.s002.doc]

**Supplemental File 1**

**Determination of the apparent rate constants of NB and SDBS in Fe^2+^/S_2_O_8_^2−^ and Fe^2+^/H_2_O_2_ systems.** Due to the fast degradation of NB (or SDBS) in the first 1 min in both Fe^2+^/S_2_O_8_^2−^ and Fe^2+^/H_2_O_2_ systems, it is rational to assume that the rate-determining step of the degradation process is the reaction of NB (or SDBS) with SO_4_•^−^ (or •OH) radicals. For the NB degradation within Fe^2+^/S_2_O_8_^2−^ system, the initial rate (*r*_0_) of the degradation reaction is estimated by Eq. 1.

(1)

where *k*′ is the rate constant, and [SO_4_•^−^] and [NB] are the concentrations of SO_4_•^−^ and NB, respectively. It is further assumed that in the initial stage of the reaction, excessive SO_4_•^−^ radicals are generated rapidly, and the concentration of SO_4_•^−^ is regarded as a constant. Therefore, Eq. 1 can be simplified to Eq. 2 and Eq. 3,

$r_{0}=k_{NB}[NB]_{0}$ (2)

$k_{NB}=r_{0}/[NB]_{0}$ (3)

where *k*_NB_ is the apparent rate constant of NB degradation. The data were analyzed by using a polynomial regression analysis and the derivative of the equation at *t* = 0 can be interpreted as being tangent to the polynomial line at t = 0 and is represented as *r*_0_, then the *k*_NB_ values were calculated according to Eq. 3. With the same method mentioned above, the apparent rate constants of NB and SDBS degradation within Fe^2+^/S_2_O_8_^2−^ and Fe^2+^/H_2_O_2_ systems were obtained.
